# Continuous positive airway pressure (CPAP) vs noninvasive positive pressure ventilation (NIPPV) vs noninvasive high frequency oscillation ventilation (NHFOV) as post-extubation support in preterm neonates: protocol for an assessor-blinded, multicenter, randomized controlled trial

**DOI:** 10.1186/s12887-019-1625-1

**Published:** 2019-07-26

**Authors:** Yuan Shi, Daniele De Luca, for the NASal OscillatioN post-Extubation (NASONE) study group, Shi Yuan, Chen Long, Xingwang Zhu, Huanhuan Li, Xiaoyun Zhong, Sijie Song, Zhang Lan, Li Li, Huiqiang Liu, Xiaomei Tong, Xiaojing Xu, Li Feng Cui, Ming Yi, Zhoujie Peng, Li Jie, Dongmei Chen, Weifeng Zhang, Xinzhu Lin, Wang Bin, Weimin Huang, Guangliang Bi, Shaoru He, Yumei Liu, Yang Jie, Gao Weiwei, Wuhua Liang, Yaoxun Wu, Xinnian Pan, Qiufen Wei, Yujun Chen, Bingmei Wei, Ling Liu, Xinghui Zheng, Ding Xu, Wang Fan, Bin Yi, Jingyun Shi, Yuning Li, Li Jiang, Chunming Jiang, Chenghe Tang, Hong Xiong, Huiqing Sun, Wenqing Kang, Dapeng Liu, Falin Xu, Xing Kaihui, Yang Ning, Fang Liu, Shaoguang Lv, Liu Hanchu, Yuan Wenchao, Rui Cheng, Shen Xian, Hui Wu, Laishuan Wang, Zhenying Yang, Zhang Xiao, Xue Jiang, Zhankui Li, Rong Ju, Wang Jin, Wenbin Dong, Ye Xiaoxiu, Benqing Wu, Zheng Jun, Tian Xiuying, Mingxia Li, Yanping Zhu, Nuerya Rejiafu, Li Long, Yangfang Li, Canlin He, Li Li, Hong Ying Mi, Liang Kun, Hong Cao, Linglin Xia, Chuanfeng Li, Zhaoqing Yin, Su Le, Yanxiang Cheng, Liping Shi, Wang Chenhong, Jiajun Zhu, Zhang Xuefeng, Xi Rong Gao, Bo Lv, Liu Chongde, Wang Xiaorong, Chen Liping, Li Lin, Zhang Chunli, Chen Jia, Qiyu Li, Lv Qin, Yanhong Li, Yong Ji, Yanjiang Chen, Jianhua Sun, Jun Bu, Danni Zhong, Zongyan Gao, Han Shuping, Xiaohui Chen, Caiyun Gao, Hongbin Zhu, Zhenguang Li, Hongwei Wu, Xiuyong Cheng

**Affiliations:** 1 Department of Neonatology, Children’s Hospital of Chongqing Medical University, Ministry of Education Key Laboratory of Child Development and Disorders, China International Science and Technology Cooperation Base of Child Development and Critical Disorders, Chongqing, 400014 China; 2 Division of Pediatrics and Neonatal Critical Care, “A.Béclère” Medical Center, South Paris University Hospitals, AP-HP, Paris, France; 3 Physiopathology and Therapeutic Innovation Unit-INSERM U999, South Paris-Saclay University, Paris, France; 4 Institute of Anesthesiology and Critical Care, Catholic University of the Sacred Heart, Rome, Italy

**Keywords:** Neonate, Noninvasive respiratory ventilation, Non invasive high frequency oscillatory ventilation

## Abstract

**Background:**

Various noninvasive respiratory support modalities are available in neonatal critical care in order to minimize invasive ventilation. Continuous positive airway pressure (CPAP) is the more commonly used but noninvasive positive pressure ventilation (NIPPV) seems more efficacious in the early post-extubation phase, although it is not clear if NIPPV may influence longterm outcomes. A recently introduced alternative is noninvasive high frequency oscillatory ventilation (NHFOV) which might be especially useful in babies needing high constant distending pressure. Preterm neonates may receive these respiratory supports for several weeks. Nonetheless, no data are available for the longterm use of NIPPV and NHFOV; few data exist on NHFOV and clinical outcomes, although its safety and suitability are reported in a number of preliminary short-term studies.

**Methods:**

We designed an assessor-blinded, multicenter, three-arms, parallel, pragmatic, randomized, controlled trial with a superiority design, investigating the use of CPAP vs NIPPV vs NHFOV during the whole stay in neonatal intensive care units in China. Since safety data will also be analyzed it may be considered a phase II/III trial. Moreover, subgroup analyses will be performed on patients according to prespecified criteria based on physiopathology traits: these subgroup analyses should be considered preliminary. At least 1440 neonates are supposed to be enrolled. The trial has been designed with the collaboration of international colleagues expert in NHFOV, who will also perform an *interim* analysis at the about 50% of the enrolment.

**Discussion:**

The study is applying the best trial methodology to neonatal ventilation, a field where it is often difficult to do so for practical reasons. Nonetheless, ours is also a physiology-driven trial, since interventions are applied based on physiological perspective, in order to use ventilatory techniques at their best. The pragmatic design will increase generalizability of our results but subgroup analyses according to predefined physiopathological criteria are also previewed trying to have some advantages of an explanatory design. Since not all clinicians are well versed in all respiratory techniques, the training is pivotal. We intend to apply particular care to train the participating units: a specific 3-month period and several means have been dedicated to this end.

**Trial registration:**

NCT03181958 (registered on June 9, 2017).

## Background

### General background

Respiratory distress syndrome (RDS) is the main cause of respiratory failure in preterm neonates, its incidence varying from ≈80% to ≈25% depending on gestational age [1]. When optimal prenatal care is provided, the best approach to treat RDS, according to several recent trials [2, 3], consists in providing continuous positive airway pressure (CPAP) from the first minutes of life using short binasal prongs or masks [4, 5], followed by early selective surfactant administration for babies with worsening oxygenation and/or increasing work of breathing. Any effort must be done to minimize the time under invasive mechanical ventilation (IMV) [6]. Nonetheless, clinical trials have shown that a relevant proportion of preterm neonates fails this approach and eventually need IMV during their hospitalization [7–9]. The duration of IMV is a well known risk factor for the development of bronchopulmonary dysplasia (BPD) - a condition associated with significant morbidity and mortality [10, 11].

To minimize the need of IMV, various noninvasive respiratory support modalities are available in neonatal intensive care units (NICU). A systematic review has shown that non-invasive positive pressure ventilation (NIPPV) reduces the need for IMV (within 1 week from extubation) more effectively than CPAP, although it is not clear if NIPPV may impact on the longterm need for ventilation, BPD or mortality [12]. The main drawback of neonatal NIPPV is the lack of synchronization, which is difficult to achieve and is often unavailable. A more recent alternative technique is noninvasive high frequency oscillatory ventilation (NHFOV) which consists on the application of a bias flow generating a continuous distending pressure with oscillations superimposed on spontaneous tidal breathing with no need for synchronization. The physiological, biological and clinical characteristics of NHFOV have been described elsewhere [13].

To date, there are few data about the use of NHFOV after extubation in preterm infants [14]. Relatively small case series or retrospective cohort studies suggested safety, feasibility and possible usefulness of NHFOV and have been reviewed elsewhere [13]. The only randomized parallel clinical trial comparing NHFOV to biphasic CPAP, in babies failing CPAP [15], has been criticized for methodological flaws and for not taking respiratory physiology into account [16]. An European survey showed that, despite the absence of large randomized clinical trials, NHFOV is quite widely used and no major side effects are reported, although large data about NHFOV safety are lacking [17]. This may be due to the relative NHFOV easiness of use but evidence- and physiology-based data are warranted about this technique.

### Need for a physiology-driven trial

The noninvasive respiratory support policy may fail for several reasons such as, for instance, apneas, upper airways obstruction, technical malfunctioning or increasing work of breathing due to worsening of parenchymal lung disorder. NHFOV might be beneficial in this latter case, that is in neonates needing lung recruitment with high distending pressure to open their lungs. This may be the case of extremely preterm, BPD-developing neonates who have increased airway resistances, while they are subjected to a deranged alveolarization and lung growth. More in general, neonates presenting with respiratory acidosis may also benefit from NHFOV.

NHFOV may be beneficial because it allows to increase mean airway pressure (Paw) avoiding gas trapping and hypercarbia, thanks to the superimposed high frequency oscillations. NHFOV also provides the advantages of invasive high frequency oscillatory ventilation (no need for synchronization, high efficiency in CO_2_ removal, less volume/barotrauma) and nasal CPAP (noninvasive interface, oxygenation improvement by the increase in functional residual capacity through alveolar recruitment). Several animal and bench studies investigated the physiology and peculiarities of NHFOV [13] and these data should be used to conduct a physiology-guided trial in order to avoid errors done in the old trials about invasive high frequency ventilation [16].

This study will be the first large trial aiming to compare the long-term use of CPAP vs NIPPV vs NHFOV in preterm neonates after surfactant replacement and during their entire NICU stay, to reduce the total need/duration of IMV. Noninvasive respiratory support is often used for several weeks, especially in extremely preterm neonates, but there are no clear data about the long-term effect of the different respiratory modalities and ours will be the first study in investigating this issue. Since there is a lack of formal data regarding NHFOV safety, some safety outcomes will also be addressed. Specific subgroup analyses will be conducted for pre-specified groups of patients who are more likely to benefit from NHFOV, according to the above-described physiological characteristics. We hypothesized that NHFOV is more efficacious than CPAP or NIPPV to reduce the need for IMV in neonates born between 25 and 32 weeks’ gestation, after their first extubation and until their final NICU discharge.

## Methods and design

### Trial design

**Fig. 1:**
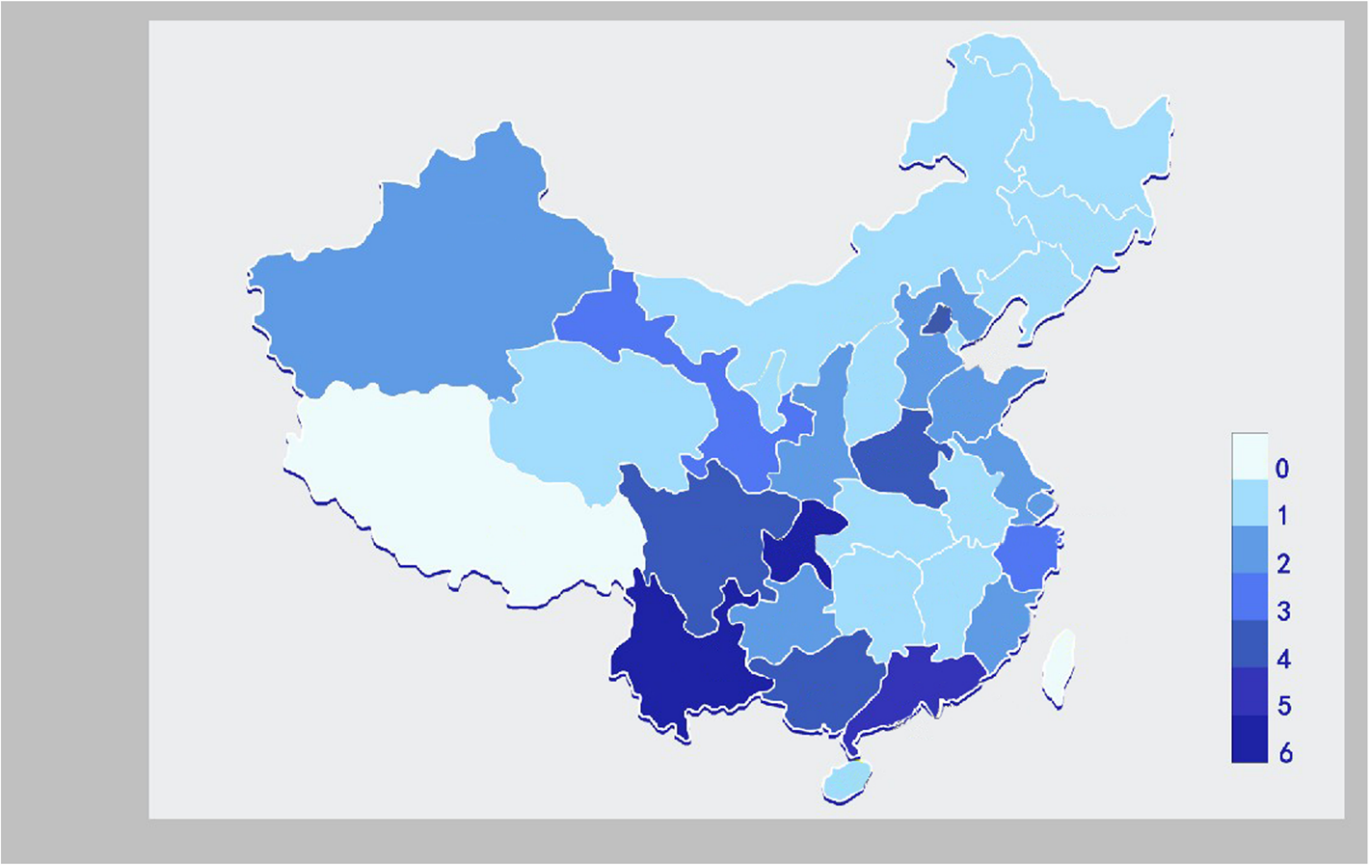
Neonatal Intensive Care Units participating to the trial. Different colors represent the number of NICU participating in each area

This will be an assessor-blinded, multi-center, three-arms, parallel, randomized, controlled trial with a superiority design, conducted in Chinese NICUs. Since safety data will also be analyzed, it may be considered the equivalent of a phase II/III trial. Since the trial will enroll all eligible patients irrespective of their lung mechanics/physiopathology and the eligibility will be judged on the basis of simple clinical data commonly used in daily NICU care, it may be defined a pragmatic trial [18]. In fact, no particular entry criteria or diagnostic procedure will be required to enroll patients; no biological samples will be collected and no additional measures will be taken. Conversely, since subgroup analyses will be performed on patients defined a posteriori according to their actual lung physiopathology, they should be considered explanatory subgroup (preliminary) analyses [18]. Results of subgroup analyses will anyway need confirmation in future, specifically designed trials. A total of 69 NICUs are included in this trial (Fig. 1). All these NICUs belong to 30 provinces or cities or autonomous regions of Chinese mainland (apart from Tibet which has been excluded for the high altitude). The trial has been designed with the collaboration of an European investigator expert in NHFOV (DLD) and will have an international expert panel performing the *interim* analysis.

### Study aim and hypothesis

Our aim is to verify the hypothesis that NHFOV is more efficacious than CPAP or NIPPV to reduce the need for IMV in neonates born between 25 and 32 weeks’ gestation, after their first extubation and until their final NICU discharge.

### Inclusion criteria

For a neonate to be included 4 criteria must be fulfilled: (1) gestational age (GA) between 25^+ 0^ and 32^+ 6^ weeks (estimated on the postmenstrual date and early gestation ultrasonographic findings); (2) assisted with any type of endotracheal ventilation; (3) post-conceptional age < 36 weeks; (4) ready to be extubated for the first time (extubation readiness requires all the following criteria: a. having received at least one loading dose of 20 mg/kg and 5 mg/kg daily maintenance dose of caffeine citrate; b. pH > 7.20 and PaCO_2_ ≤ 60 mmHg (these may be evaluated by arterialized capillary blood gas analysis or appropriately calibrated transcutaneous monitors [19] – see Appendix. Venous blood gas analysis cannot be used); c. Paw of 7–8 or 8–9 cmH_2_O, in conventional and oscillatory ventilation, respectively [6]; d. FiO_2_ ≤ 0.30; e. sufficient spontaneous breathing effort, as per clinical evaluation [20]).

### Exclusion criteria

Neonates who never needed intubation and IMV are not eligible for the study; similarly, a neonate randomized but never extubated is not eligible in the study. Moreover, neonates with at least one of the following criteria are also not eligible: (1) major congenital anomalies or chromosomal abnormalities; (2) neuromuscular diseases; (3) upper respiratory tract abnormalities; (4) need for surgery known before the first extubation; (5) Grade IV-intraventricular hemorrhage (IVH) [21] occurring before the first extubation; (6) birth weight < 600 g; (7) suspected congenital lung diseases (such as genetic anomalies of surfactant metabolism) or malformations (such as cystic adenomatous malformations, sequestration, diaphragmatic hernia) or pulmonary hypoplasia. More details are available in the appendix.

### Randomization

**Fig. 2:**
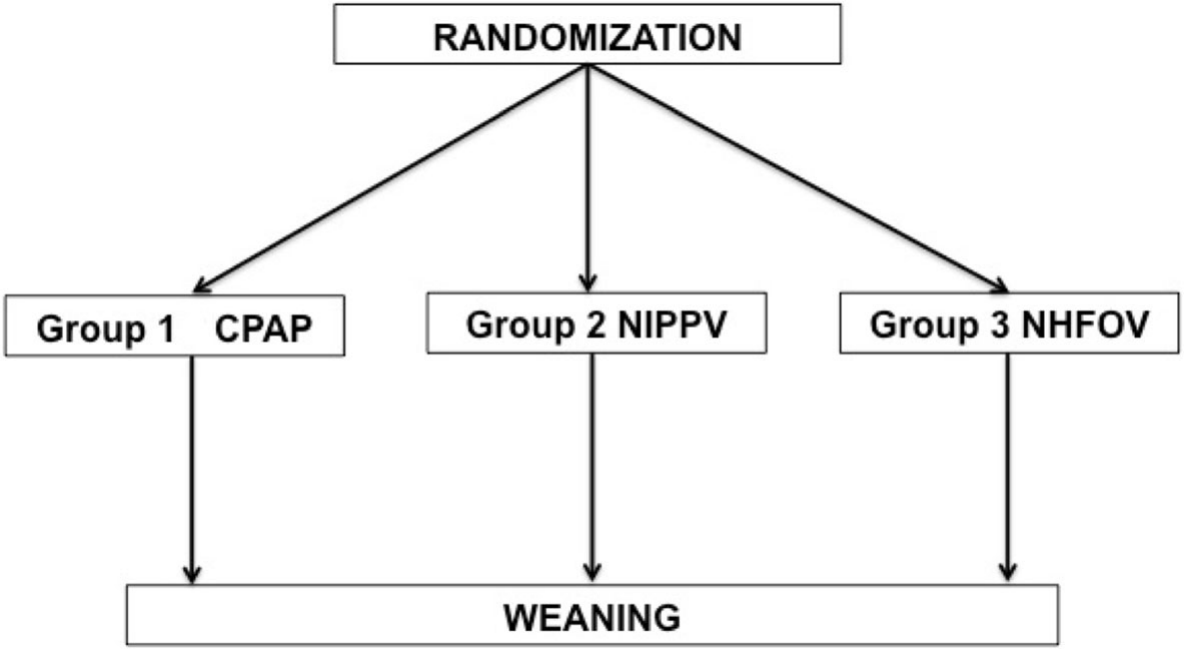
Basic study design. Neonates will stay on the assigned intervention until the final weaning. No cross-over allowed. In case of intubation, when the baby will be extubated, he will receive again his original treatment according to randomization

Neonates will be randomized and assigned either to CPAP, NIPPV or NHFOV arms with a 1:1:1 ratio, when patients fulfil all inclusion criteria and extubation is deemed imminent (anyway within 1 h). Randomization cannot be done earlier. Simple randomization will be done according to a computer-generated random number table and will be posted in a specific secured website 24/7 available. Twins will be allocated in the same treatment group. Infants randomized to one arm cannot crossover to the other or vice-versa during the study. Patients will remain under the assigned respiratory support until the weaning criteria will be met (see below). In case of intubation, when the baby will be extubated, he will receive again his original treatment according to randomization. This is summarized by Fig. 2.

### Blinding

Blinding towards the caregivers is impossible and blinding towards the patients makes no sense. However, outcomes’ assessors will be blinded, as endpoints will be recorded by assessors not involved in patients’ care: they will review patients’ files masked for the type of treatment. An assessor per each participating NICU will be nominated. Moreover, investigators performing the final statistical analyses will also be blinded to the treatment allocation.

### Primary outcomes

The primary outcomes will be: (1) duration of IMV (in days) from the randomization; (2) ventilator-free days (calculated as described in the appendix); (3) the number of reintubation. Neonates will be re-intubated if one of the following occurs:

- severe respiratory acidosis (defined as PaCO_2_ > 65 mmHg with pH < 7.2);
- hypoxia refractory to study intervention (defined as need for FiO_2_ > 0.4 to obtain SpO_2_ < 90%, with and maximal pressures allowed in the study arm – see below) for at least 4 h;
- severe apnea (defined as recurrent apnea with > 3 episodes/h associated with heart rate < 100/min or a single episode of apnea requiring bag and mask ventilation, or associated with SpO_2_ < 85% and FiO_2_ > 0.6);
- pulmonary hemorrhage (defined as brightly blood tracheal secretion associated with sharp increase in oxygen and Paw and with the occurrence of “white lungs”, new infiltrates or consolidations at the chest X-rays or lung ultrasound);
- severe respiratory distress (defined as Silverman score > 4) for at least 4 h;
- hemodynamic instability, defined as mean arterial pressure < 10th percentile of appropriate nomograms [22, 23] or anyway need of dopamine (if > 5 mcg/Kg/min) or dobutamine (if > 5 mcg/Kg/min) or any dose of noradrenaline, adrenaline, milrinone, nitric oxide or other pulmonary vasodilators.
- cardio-respiratory arrest.

### Secondary outcomes

The secondary outcomes will be: (1) airleaks (pneumothorax and/or pneumomediastinum) occurred *after* the extubation; (2) BPD, defined according to the NICHD definition [24]; (3) hemodynamically significant patent *ductus arteriosus* (PDA), defined according to local NICU protocols; (4) retinopathy of prematurity (ROP) > 2nd stage [25]; (5) necrotizing enterocolitis (NEC) ≥ 2nd stage [26]; (6) IVH > 2nd grade [21]; (7) need for postnatal steroids; (8) in-hospital mortality; (9) composite mortality/BPD; (10) weekly weight gain (in grams/d) for the first 4 weeks of life or until NICU discharge, whichever comes first.

### Safety outcomes

The safety outcomes will be the following: (1) weekly number of vomiting/d; (2) weekly volume of gastric residual (ml/d); (3) weekly number of apneas/d; (4) nasal skin injury (weekly defined by a clinical score [27] as: 0 (zero, absence of injury), stage I (non-blanching erythema), stage II (superficial erosion), stage III (necrosis of full thickness of skin) – more details in the appendix). These outcomes will be averaged over each week for the first 4 weeks of life or until NICU discharge, whichever comes first. Finally, (5) Premature Infant Pain Profile (PIPP) score [28] will be considered (averaged from values available in the first 48 h from the allocation to CPAP, NIPPV or NHFOV). Investigators will also record any serious adverse event (defined as an untoward medical occurrence that is believed by the investigators to be causally related to study-intervention and results in any of the following: life-threatening condition (that is, immediate risk of death); severe or permanent disability, prolonged hospitalization).

### Study intervention

When the neonate had fulfilled the extubation criteria, this latter will take place with a gentle intratracheal suction, following local clinical policies. Upper airways will then be suctioned and intervention will be started immediately as follows:

#### Ventilators

- **CPAP**: CPAP will be provided by either variable flow or continuous flow devices, as there is no clear evidence that one type of CPAP generator would be better than any other [29].
- **NIPPV**: NIPPV will be provided by any type of neonatal ventilator able to generate enough pressure according to the protocol (see below). Synchronization will not be applied, as many currently marketed neonatal ventilators do not provide it for NIPPV [30].
- **NHFOV**: NHFOV will only be provided with piston/membrane oscillators able to provide an active expiratory phase (that is, Acutronic FABIAN-III, SLE 5000, Loweinstein Med LEONI+, Sensormedics 3100A). Other machines pretending to provide high frequency ventilation using other technologies will not be used.

Before the beginning of the study all ventilators will be checked to ensure that there is no malfunction.

#### Interfaces

CPAP, NIPPV and NHFOV will be administered through short, low-resistance binasal prongs and/or nasal masks, since these are supposed to be the best in terms of resistive charge and leaks [4, 5]. Nasal prongs size will be chosen according to the nares’ diameter as the best fitting ones (the largest ones fitting the nares without blanching the surrounding tissues) and following manufacturer’s recommendations. Nasal masks will also be appropriately sized according to manufacturer’s recommendations. Alternating masks and prongs, according to clinical evaluation, is allowed in order to reduce the risk for nasal skin injury. Particular care (e.g.: pacifiers, positioning, nursing) will be applied to reduce leaks and improve patients’ comfort. These latters will be evaluated through a dedicated 30′ observation period when study the intervention will be instigated. Non-pharmacological sedation with pacifiers and 33% glucose solution will be provided, when needed; no other sedation will be allowed. RAMCannula® are not allowed in the trial due to their resistive charge and their relevant pressure leaks [31, 32].

#### Ventilatory management

The three interventions will be managed as follows:

- **CPAP**: Neonates assigned to the CPAP group were initiated on a pressure of 5 cmH_2_O. CPAP can be raised in steps of 1 cmH_2_O up to 8 cmH_2_O. If this is not enough to maintain SpO_2_ between 90 and 95%, FiO_2_ will be increased up to 0.40.
- **NIPPV**: neonates assigned to the NIPPV group will be started with the following parameters: a) positive end-expiratory pressure (PEEP) of 4 cmH_2_O (can be raised in steps of 1 cmH_2_O to max 8 cmH_2_O, according to the oxygenation). b) Peak Inspiratory Pressure (PIP) of 15 cmH_2_O (can be raised in steps of 1 cmH_2_O to max 25 cmH_2_O, according to oxygenation, PaCO_2_ levels and the chest expansion); maximal allowed FiO_2_ will be 0.40 and SpO_2_ targets will be 90–95%. c) inspiratory time (IT) will be 0.45–0.5 s (according to clinicians’ evaluation of leaks and the appearance of the pressure curve: a small pressure plateau is required and flow may be set accordingly) and rate will be started at 30 bpm (can be raised in steps of 5 bpm to max 50 bpm, according to PaCO_2_ levels).
- **NHFOV**: neonates assigned to NHFOV will be started with the following boundaries, according to available physiological and mechanical data, as suggested elsewhere [13]: a) Paw of 10 cmH_2_O (can be changed in steps of 1 cmH_2_O within the range range 5-16 cmH_2_O); Paw will be titrated (within the range) according to open lung strategy, performing alveolar recruitment, similar to what is done in invasive high frequency oscillatory ventilation targeting a FiO_2_ ≤ 25–30%, as published elsewhere [33]. Maximal allowed FiO_2_ will be 0.40 and SpO_2_ targets will be 90–95%. b) frequency of 10 Hz (can be changed in steps of 1 Hz within the range 8-15 Hz). c) Inspiratory time 50% (1:1) [34]. d) amplitude 25 cmH_2_O (can be changed in steps of 5 cmH_2_O within the range 25–50 cmH_2_O) [34, 35]; amplitude will be titrated according to PaCO_2_. It is not strictly necessary to have visible chest oscillations, as PaCO_2_ elimination during NHFOV also occurs in the upper airway dead space [36]. In case of hypercarbia, amplitude will be increased first and then frequency will be lowered (within the above-described ranges), however, if nasal masks are used, the amplitude should be kept at the maximum and PaCO_2_ controlled by frequency titration, as oscillation amplitude using masks is more dampened [37, 38].

### Monitoring and concurrent treatments/diagnostic measures

PaCO_2_ will be measured using arterialized capillary blood gas analysis and/or transcutaneous monitoring according to local policies. Transcutaneous monitoring will be performed according to the American Association of Respiratory Care guidelines [19] and the manufacturer’s recommendations. Frequency of blood gas analysis will be decided by the attending clinicians. All neonates will be continuously monitored for SpO_2_, ECG, heart and respiratory rate. To avoid abdominal distention, a feeding tube will be placed in the stomach through the mouth and gas will be periodically aspirated according to nurses’ evaluation in all study arms. Moreover, the following treatments or tests will be provided:

- Heart ultrasound to evaluate cardiac morphology, pulmonary pressures and PDA, within the first 3d of life and subsequently repeated, if needed.
- Cerebral ultrasound within 48 h of life and weekly thereafter, until discharge, if needed.
- Routine measures to prevent BPD; routine fluid/nutritional policy; routine caffeine therapy.
- Placement of umbilical central venous catheter and/or peripherally inserted central venous lines. Placement of arterial lines if needed, according to local policies.
- Routine therapies according to local policies (i.e.: antibiotics, PDA closure drugs…).

In general, routine medical care and nursing will not be changed because of the study, out of the trial intervention; the clinical care will be identical in the three study arms. No additional blood samples are required solely for study purposes.

### Weaning from study interventions

The study intervention will be progressively weaned, according to clinical evaluation and respecting the following guidelines:

- ◦ in the CPAP arm, pressure will be reduced by 1 cmH_2_O steps down to a minimum of 3 cmH_2_O;
- ◦ in the NIPPV arm, PIP and PEEP will be reduced by 1 cmH_2_O steps down to a minimum of 5 and 3 cmH_2_O, respectively. Similarly, frequency will be reduced to a minimum level of 20 bpm in steps of 5 bpm.
- ◦ in the NHFOV arm, amplitude will be reduced to the minimum initial level of 20 cmH_2_O and Paw will be reduced by 1 cmH_2_O down to a minimum of 3–5 cmH_2_O (depending on the ventilator used).

The study intervention (CPAP, NIPPV or NHFOV) will be stopped when the above-described minimum parameters are reached and maintained for at least 48 h with the following: **(1)** FiO_2_ ≤ 0.25; **(2)** Silverman score < 3; **(3)** no apneas or bradycardia without spontaneous recovery. If a baby will desaturate (SpO_2_ < 85% with FiO_2_ > 25%) or has relevant dyspnea (Silverman≧3) or more than 3 apneas/d, the intervention (CPAP, NIPPV or NHFOV) will be restarted for at least 48 h and then re-evaluated. The end of study intervention may occur at any time during hospitalization if the above described criteria are met. When study interventions end, the neonate may be placed under low flow oxygen therapy (max 1 L/min), if needed, according to clinicians’ evaluation and local protocols. Anyway, when a post-conceptional age of 36 weeks is reached, if the patient still needs noninvasive respiratory support, he/she will be shifted to CPAP and managed according to clinical evaluation and local policies.

### End of the study

A patient may exit from the study for any of the following reasons:

1. Death.
2. In any case, when the 36 weeks’ post-conceptional age is reached.
3. If parents or guardians withdraw an already given consent for the participation (in that case the patient will keep receiving the whole routine clinical care; data acquired will be immediately destroyed).

### Training

Since not all clinicians are well versed in all respiratory techniques and, particularly, NHFOV is a relatively new technique, the training is capital for the trial success. The protocol will be diffused between participating centers at least 3 months before the study begins. One investigator (YS) will explain the study protocol in an in-person meeting with all investigators. During the 3 months, clinicians will familiarize with the protocol and the respiratory techniques and an expert in NHFOV (DLD) will be available to solve any doubt. A dedicated social media chat has been set to facilitate these contacts. Moreover, an expert in NHFOV (DLD) will conduct, site visits and a webinar teaching about the technique and will personally visit some participating NICUs.

### Sample size calculation

It is difficult to calculate a sample size, since this is the first trial to investigate CPAP vs NIPPV vs NHFOV in post-extubation phase in preterm babies. However, a previous prospective, cohort, non-randomized, pilot study comparing post-extubation NIPPV and NHFOV in preterm neonates provided data about the primary outcome “duration of mechanical ventilation”. This preliminary study showed a reduction of ≈30% for babies receiving NHFOV, as compared to those treated with NIPPV, but it has been presented only as abstract so far [39]. A randomized trial of NIPPV vs CPAP by Ramanathan et al. showed a similar reduction [40]. Since these trials have not the same design of ours, we decide to be more prudent and we aimed a difference of 20% in the duration of mechanical ventilation. Considering an alpha-error of 0.05 (with a Bonferroni correction at 0.017) and a power of 95%, 480 neonates should be enrolled in each arm (with a 1:1:1 design). Thus, a total of at least 1440 neonates will be enrolled. Sample size calculation has been performed with GPower3.1.9.3 [41]. We do not foresee any problem in reaching this sample size over 12–18 months, given the large network and the number of potentially eligible neonates.

### Data collection

All data can be obtained from the clinical notes. Data will be recorded in real time on web-based case report forms provided by OpenCDMS. The website will be tested with fictitious data before the actual enrolment. Data will be entered by an assessor per each center. Assessors will be research nurses or local investigators blinded to the study intervention and not directly involved in patients’ care. Access to the form will be secured and patients will be de-identified. Clinical information will be collected at the following time-points:

1. **Before the intervention begins:** information on eligibility; baseline clinical informations, respiratory diagnosis, critical risk index for babies-II (CRIB-II) score [41].
2. **Following study intervention:** ventilatory parameters, SpO_2_, blood gas values before the extubation if available. PaO_2_, PaCO_2_, SpO_2_ and pH between 6 h and 24 h from the extubation.
3. **Follow-up:** NICU length stay, duration of IMV, number of reintubation, ventilator free days, duration of oxygen therapy, duration of the study intervention (CPAP, NIPPV or NHFOV), airleaks, PDA, BPD, ROP >2nd stage, NEC ≥ 2nd stage, IVH > 2nd grade, need for postnatal steroids, in-hospital mortality, composite mortality/BPD, weekly weight gain (in grams/d) for the first 4 weeks of life or until NICU discharge, whichever comes first. Moreover, the following safety data will be recorded: weekly number of vomiting/d, weekly volume of gastric residual (ml/d); weekly number of apneas/d; nasal skin injury (weekly defined by a 1–2-3 clinical score [27]). These outcomes will be averaged over each week for the first 4 weeks of life or until NICU discharge, whichever comes first. Finally, PIPP score [28] will be recorded in the first 48 h from the allocation. Abdominal circumference at 48 h and 96 h from the instigation of CPAP, NIPPV or NHFOV will also be recorded.

### Statistics

Data analysis will be performed blindly to the type of treatment received. An *intention-to-treat* analysis will be applied. An *interim* analysis will be performed at approximately 50% of the enrolment together by international expert panel. First, data will be checked for normality using Kolmogorov-Smirnov test and results will be presented as odds ratio (OR) and 95% confidence interval (CI) or adjusted OR and 95%CI, and mean ± standard deviation or median [quartiles], as appropriate.

Univariate logistic or linear regressions will be performed, according to the type of variable, as appropriate. Univariate Cox’s proportional regression will be used for mortality and the duration of IMV. Multivariate regressions will also be performed for selected outcomes, if needed (that is, if a baseline characteristic differs between the two arms with a *p* < 0.2 at the univariate analysis the results will be adjusted for that variable). For each multivariate analysis, multicollinearity will be previously checked considering condition index of Eigenvalues and Variance Inflation Factor [43, 44]. *p*-values < 0.05 will be considered statistically significant.

The following sub-group analyses will be performed:

1. Analysis for babies ≤28 weeks’ gestation.
2. Analysis for babies who have been invasively ventilated for at least 1 week from birth.
3. Analysis for babies with PaCO_2_ > 50 mmHg before the extubation or at the 6 h or 24 h after extubation.

These subgroup analyses have been chosen trying to give more insight about the use of the trialed respiratory techniques for patients with different respiratory physiopathology, as there is no doubt that a more preterm baby have a higher risk of reintubation or that those stacked on the ventilator have “evolving BPD”. However, subgroups have been selected using simple clinical data to remain pragmatic and facilitate the recruitment.

### International colleagues’ panel

This panel will analyze all data in an *interim* analysis at approximately 50% of the trial enrolment. The panel will be composed by international neonatologists or pediatric intensivists experts in respiratory care. The risk for patients’ safety is estimated as very low, since the interventions are known and already used in NICU care. The panel will analyze all data outcome, both on efficacy and safety. This is unusual in a trial about neonatal ventilation but will help to increase the quality of data. Unless this happens, however, the investigators will remain ignorant of the *interim* results. The board will advice the principal investigator (YS) who will remain the only responsible for the trial conduction and for any eventual decision to stop or continue it. The board will also give some advices on quality of data and their analysis: the principal investigator will remain responsible for the final data, analysis and study results. The board members will be completely independent from the trial, not working at any institution enrolling patients and with no direct conflict of interest.

## Discussion

### On the trial methods and limitations

Ours is a study trying to apply the methodology of pharmacological trials to neonatal mechanical ventilation, a field where it is often difficult to do so for practical reasons (blinding difficulties, long study times, difficult recruitment, lack of funding). Nonetheless, ours is also a “physiological” trial, since interventions are applied based on physiological perspective, in order to optimize the use of the different ventilatory techniques. This has been often neglected in the past, but we are exploiting the previous bench and in vivo mechanical data [13] in order to optimize the use of NHFOV. Therefore, we hope to provide high quality data, although further trials may be required, as this is only the first large one on NHFOV and some questions will remain unanswered (see below).

Moreover, we applied a pragmatic design in order to increase generalizability of our results [18]. This means enrolling many babies in the common daily NICU care, irrespective of their actual mechanical and biological lung condition, without any particular test prior to enrolment. However, an explanatory design may be more appropriate for a recently introduced new therapeutic intervention [44, 45], since a pragmatic design may lead to dilute the effect of the interventions and mask their effect on a particular type of patients. This is the reason why we also previewed three subgroup analyses according to predefined criteria in order to identify patients (a posteriori) for some of their characteristics. This has been possible because the large population size will allow us to recruit a certain number of patients with homogeneous traits and try to have some advantages of the explanatory design in the subgroup analyses [18]. However, results of these subgroup analyses, even if promising, must be considered preliminary and will unavoidably require further confirmation in subsequent specifically designed trials. Alternatively, future trials using more stringent criteria, and/or also using less common monitoring techniques, will be useful to better describe lung physiopathology and recruit patients with a fully explanatory design.

We cannot clarify if increasing CPAP levels may eventually approach results obtained with NIPPV or NHFOV used with higher mean airway pressure. This could be considered as a trial limitation, albeit we must consider that:

- the use of higher CPAP levels is controversial and not standardized. An open lung strategy during CPAP has never been formally studied while this has been done in invasive HFOV [33]. European guidelines do not advice to use higher CPAP levels [6, 46]. Anyhow, we could not change this point, as higher CPAP levels are not common standard of care in Chinese NICU and the trial needed to respect this in order to be pragmatic.
- Increasing CPAP level without any real ventilation, may increase the risk of gas trapping (even if this may not be clinically evident as airleak syndrome). The risks or consequences related to this might not be accurately detected in a study on non-invasive respiratory support where patients are not closely monitored as those intubated or those enrolled in explanatory trials using particular techniques looking at lung volume (for instance, electrical impedance tomography [47], respiratory inductance pletysmography [48], or semi-quantititative lung ultrasound [49]).
- The presence of oscillatory pressure waveform will have an effect only on PaCO_2_ levels, as previously by demonstrated by basic physiology [13, 36] and meta-analysis of NHFOV clinical trials published so far [50]. This is the reason why we included a subgroup analysis for hypercarbic babies in order to detect as much as possible the potential effect of this ventilatory trait.
- If allowing higher mean airway pressures in NHFOV than in CPAP may influence the interpretations of results, this is also true for NIPPV, which allows increasing mean airway pressures, while providing a real ventilation by using a conventional volume delivery. We believe that this may be a main reason behind the lack of diffusion and standardization of higher levels CPAP policies, whereas techniques actually providing a true ventilation are felt safer from a physiopathological point of view.

Thus, we are interesting and willing to see if NHFOV is able to provide advantages on CPAP (used with common pressure levels), due to its global technical characteristics, including the possibility to increase constant distending pressure preventing the development of airleaks with the application of the oscillatory pressure waveform.

Finally, we do not allow any cross-over between the study arms for the following reasons. While there are some data indicating possible efficacy of NIPPV to avoid re-intubation, this evidence is generally considered of moderate-low quality [12]. Moreover, the general absence of synchronization is reducing the efficacy and creating an unjustified difference with NIPPV provided in critically ill children and adults. As such, European guidelines do not clearly suggest the use of NIPPV, unless it is synchronized [46], and there is no clear guidliens on this issue from the American Academy of Pediatrics. Therefore, we decided not to allow the cross-over and choose directly to the best evidenced and more widely used rescue practice which remains endotracheal intubation and IMV. This is also in agreement with the pragmatic study design.

### Ethical considerations

This trial is worth to be conducted given the uncertainty about the superiority of one respiratory technique over the others, especially for babies at highest risk. Moreover, NHFOV might be actually superior to the other techniques, as we may hypothesize this from the currently available data [13]. NHFOV has been already studied in preliminary cross-over trials, in bench and animal studies [13], while invasive HFOV is often used for severe respiratory failure. NIPPV has been studied in several randomized controlled trials enrolling smaller population and/or without triple comparison against CPAP and NHFOV [12]. Thus, the tested interventions are not totally new and there is a great drive towards noninvasive ventilation in NICU care: this study is a new step within this framework.

Thus, the risks for babies are minimized and the monitoring will quickly report any possible problem. Out of the studied intervention, the participation to the study will not change the routine clinical assistance. Data will be anonymously recorded, secured and accessible only to the investigators and to the parents/guardians. In no case the recorded data will be used for purposes out of those specified in the trial protocol. Moreover, the trial is only funded by a public Chinese research program, thus it will not have external industrial influences and has the merit to try filling the lack of public funding for neonatal ventilation trials [51].

### Publication policy

Study results will be presented to each investigator by teleconference and/or e-mail by the principal investigator (YS). If possible an investigator meeting in occasion of one of the major congresses in the field of pediatrics or critical care (the European Society for Pediatric Research or European Society for Pediatric and Neonatal Intensive Care Congresses or the Pediatric Academic Societies Meeting) will be organized. Data will be also partially presented at these meetings. Abstracts and manuscripts will be circulated between all investigators for revision and will be approved by all authors in their final version. All manuscripts will be authored by a group authorship: full results will be published in a major journal in the field of general medicine or pediatrics or critical care. Authorship criteria of the International Committee of Medical Journal Editors will be followed.

## Appendix

### Clarifications for exclusion criteria

Neonates who never needed intubation and IMV are not eligible for the study; similarly, a neonate randomized but never extubated is not eligible. This means that, if a randomized neonate is not actually extubated within 1 h, because there has been a worsening of his conditions or death, he is excluded from the study. Randomization must be done as close as possible to the extubation, once all inclusion criteria are fulfilled (see above), and anyway within 1 h from the actual extubation.

Some exclusion criteria are represented by congenital disorders. When a patient is affected by these disorders his biology and physiology are significantly deranged: they are not eligible and will not be randomized. If the condition has been discovered/suspected after the randomization but before the study inclusion (that is, before extubation), they will not receive the study intervention and will not entry in the study. If one of these conditions is diagnosed after the inclusion in the study, the neonate will be excluded a posteriori. This is the case of neonates with major congenital anomalies, chromosomal abnormalities, neuromuscular diseases, congenital upper respiratory tract abnormalities and congenital lung diseases or malformations or hypoplasia. Examples of these conditions are: genetic syndromes, surfactant protein deficiency, congenital adenomatous pulmonary malformations, congenital diaphragmatic hernia or sequestration, congenital hypoventilation syndrome, pulmonary hypoplasia or any metabolic disease. Same applies for the need for surgery anticipated antenatally or before the first extubation, as this is usually related to congenital malformations. These neonates are not eligible and will not be randomized. If the condition has been discovered/suspected after the randomization but before the study inclusion (that is, before extubation) they will not receive the study intervention and will not entry in the study. If a surgery will be needed later during the NICU hospitalization for other reasons (for instance for PDA ligation or NEC), the patient will regularly continue the trial. These needing surgery conditions will be noticed amongst the outcomes.

Grade IV-IVH known before the first extubation is a significant risk factor for prognosis and for quality of life. Continuing the NICU care in this situation may be considered unethical, depending to different local settings, cultures, ethical and religious beliefs. This may significantly impact on the trial outcomes. These neonates are not eligible and will not be randomized. If grade IV-IVH has been discovered/suspected after the randomization but before the study inclusion (that is, before extubation), they will not receive the study intervention and will not entry in the study. If Grade IV-IVH will be diagnosed after the study inclusion, the patient will continue the study regularly and this will be noticed amongst the outcomes.

### List of study definitions/assessments (in alphabetical order)

#### Antenatal steroids

Antenatal steroid prophylaxis will be considered complete if two 12 mg-doses of betamethasone 24 h apart and between 1 day and 7 days before the delivery had been given.

#### Blood gas analysis

Blood gas values may only be obtained in following three ways (venous blood gas analysis is not allowed in the study).

- ***Arterial blood from indwelling arterial lines,*** if one of these was placed for clinical reasons. As these are likely to be unavailable in the majority of cases, the following two alternative techniques may be used. Blood gas analysis will be obtained upon attending neonatologist decision.
- ***Arterialized capillary blood*** gas analysis is performed, warming a patient’s heel for 10′ and collecting 200 μL of blood into a heparinized micro-tube. This must be analyzed by a blood gas analyzer within 5′. Blood gas analysis will be obtained upon attending neonatologist decision.
- ***Transcutaneous blood gas monitoring*** will be performed according to American Associations of Respiratory Care guidelines [19] and the device manufacturer’s recommendations and using an electrode temperature of 44 °C for a short time (max 10-15 min). Particular care must be provided to avoid skin injury in extremely preterm neonates: in some cases, a temperature of 42 °C may be more suitable. Transcutaneous gas measurements will be obtained upon attending neonatologist decision.

**Fig. 3:**
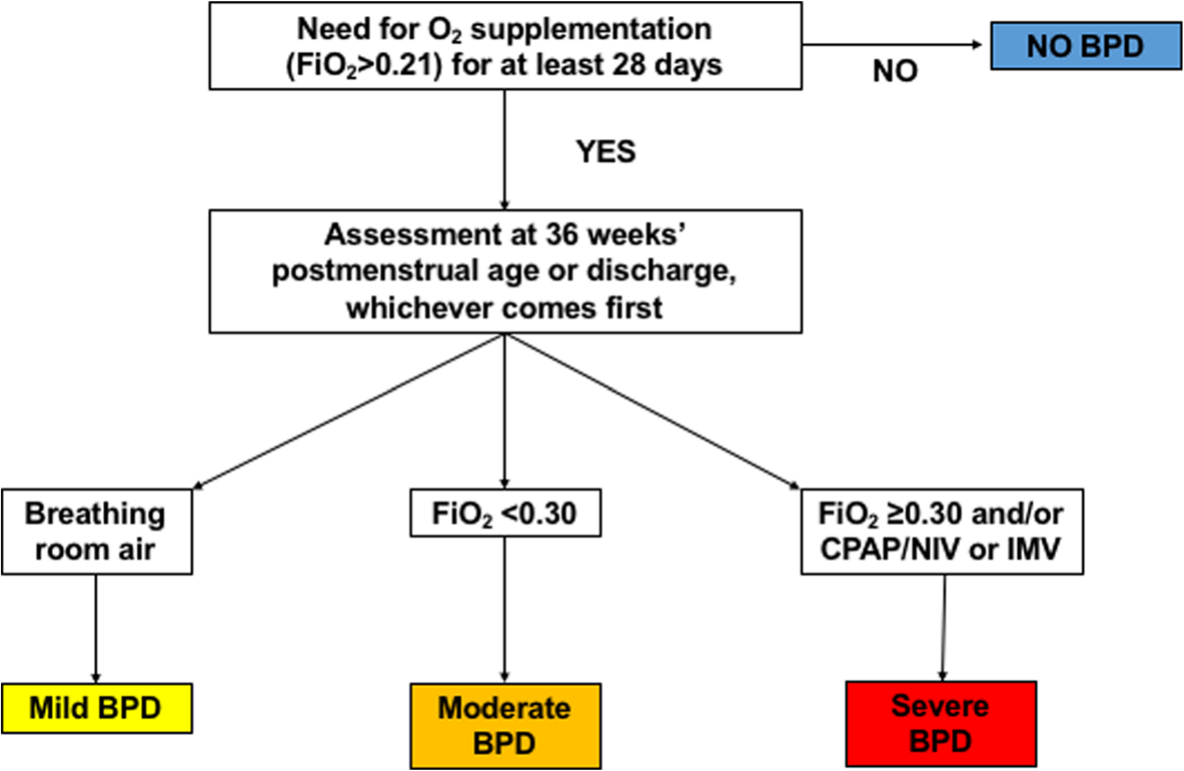
Current bronchopulmonary dysplasia definition [24]. The figure represents the algorithms to apply the definition only in babies ≤32 weeks’ gestation, as these are those enrolled in the trial

**BPD definition** [24] for neonates ≤32 weeks’ gestation, as described in Fig. 3.

**Clinical Risk Index for Babies (CRIB-II) score** [42]. This is an estimator of the clinical severity at the NICU admission. CRIB-II score considers 4 variables: birth weight, GA, base excess within the 1^st^h of life and temperature at the admission*.* An online tool will be used to calculate the score.

**Time on CPAP/NIPPV/NHFOV.** Number of days spent under these respiratory supports will be registered and rounded to the closest entire number.

**Gestational age (GA).** GA is determined based on last menstrual period or early ultrasound scan (within the first trimester). If a discrepancy of more than 2 weeks exists, the early ultrasound scan will be chosen.

**Nasal injuries.** These are classified by using a clinical score [26] as stage I (non-blanching erythema), stage II (superficial erosion), stage III (necrosis of full thickness of skin) in the skin area in contact with nasal prongs, as described elsewhere [27]. The score will be 0 (zero), in case of absence of any injury.

**Premature Infant Pain Profile (PIPP)** score, as described elsewhere [28].

**Pulmonary hypoplasia.** This will be clinically defined if anamnestic (prenatal findings: small lung volume), imaging (diffuse chest x-ray opacity or hypo-density) and clinical data (extremely low gestational age, olygo-anhydramnios, severe pulmonary hypertension, refractory hypoxia) are present. Pulmonary hypoplasia usually does not allow survival.

**Respiratory main diagnosis.** A respiratory main diagnosis that required IMV (±surfactant administration) has to be given according to the following criteria. **RDS**: respiratory distress appearing within the first 24 h of life, with complete, sustained, and prompt response to surfactant or lung recruitment or both; additional non-mandatory criteria are lung imaging (chest X-rays or ultrasound, according to local policies) supporting the diagnosis or lamellar body counts≤30 000/mm^3^, or both [52]. **Pneumonia**: broncho-alveolar lavage fluid or blood positive culture or C-reactive protein and/or procalcitonin beyond the normal values, together with radiological signs of infection(infiltrates and/or consolidation and/or loss of aeration) [53].

**Sepsis (international pediatric sepsis definition)**: presence of systemic inflammatory response syndrome (SIRS) together with a suspected or proven (by positive culture, tissue stain, or polymerase chain reaction test) infection caused by any pathogen or a clinical syndrome associated with a high probability of infection [54]. Infection is suspected according to anamnesis, clinical exam, imaging or laboratory tests. Evidence of SIRS is given by the presence of at least two of the following four criteria, one of which must be abnormal temperature or leukocytes:

- ▪ Core temperature > 38.5 °C or < 36 °C.
- ▪ Tachycardia, defined as a mean heart > 180 bpm in the absence of external stimulus, chronic drugs, or painful stimuli; or otherwise unexplained persistent elevation over a 0.5- to 4-h time period OR bradycardia, defined as a mean heart rate < 100 bpm in the absence of external vagal stimulus, −blocker drugs, or congenital heart disease; or otherwise unexplained persistent depression over a 0.5-h time period.
- ▪ Mean respiratory rate > 60/min or need for IMV for an acute process not related to underlying neuromuscular disease or the receipt of general anesthesia.
- ▪ Leukocyte count elevated or depressed or 10% immature neutrophils.

**Meconium aspiration syndrome (MAS):** Presence of meconium-stained amniotic fluid and secretions upon tracheal suctioning with onset of respiratory distress early from birth and chest X-rays or lung ultrasound typical for MAS [55]. **Neonatal ARDS:** defined as per the international Montreux definition [52].

**Ventilator free days** defined as the number of days spent in the NICU without IMV. One point is given for each day during the NICU stay that patients are both alive and free of mechanical ventilation; in case of death, zero days will be given [56].

### Guidelines for protocol preparation

**Fig. 4:**
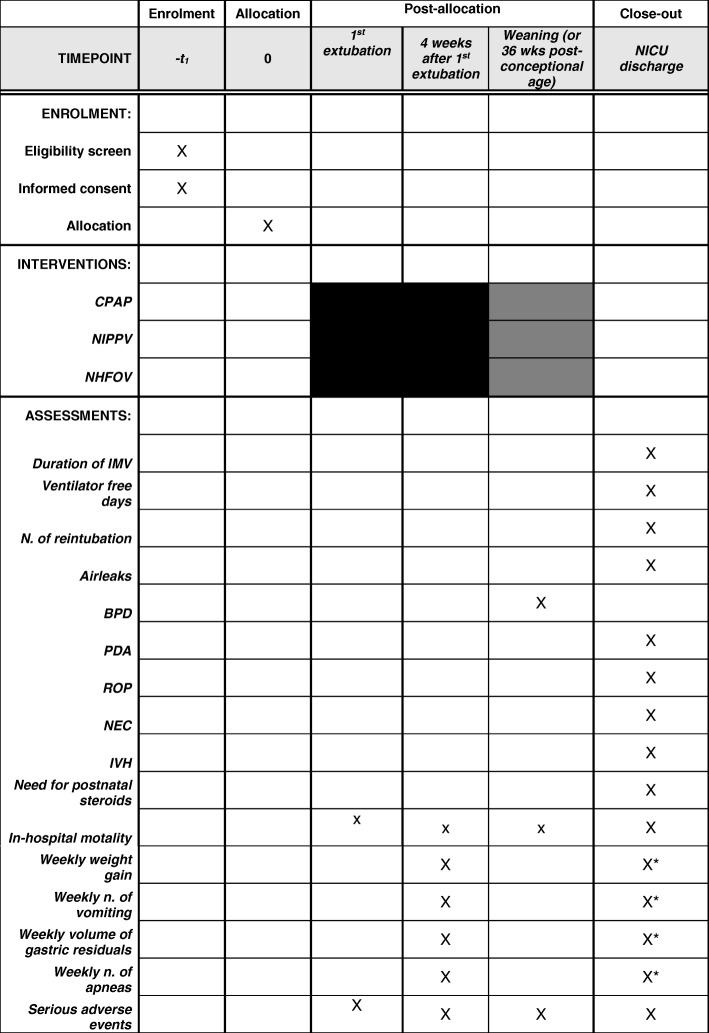
Trial flow-chart according to SPIRIT guidelines [57]. Black squares indicate timepoints when the intervention will surely be provided, while grey squares indicate a time point (36 weeks’ postconceptional age) when the intervention may be provided unless it has been interrupted earlier. All assessments will be performed at the NICU discharge apart from the diagnosis of BPD (that requires evaluation of at 36 weeks’ postconceptional age [24]), safety outcomes and serious adverse events

The protocol has been prepared according to Standard Protocol Items: Recommendations for Interventional Trials (SPIRIT) guidelines [57], adapting them as much as possible to the field of neonatal ventilation trials. Figure 4 illustrates SPIRIT guidelines for our protocol.

## Data Availability

Not applicable for the study protocol. The datasets used and/or analyzed during the trial will be made available from the corresponding author on reasonable request.

## Abbreviations

ARDS: Acute respiratory distress syndrome
BPD: Bronchopulmonary dysplasia
CI: Confidence interval
CPAP: Continuous positive airway pressure
CRIB-II: Critical risk index for babies-II
GA: Gestational age
IMV: Invasive mechanical
IVH: Intraventricular hemorrhage
MAS: Meconium aspiration syndrome
NEC: Necrotizing enterocolitis
NHFOV: Noninvasive high frequency oscillatory ventilation
NICU: Neonatal intensive care unit
NIPPV: Noninvasive positive pressure ventilation
OR: Odds ratio
Paw: Mean airway pressure
PDA: Patent *ductus arteriosus*
PIPP: Premature Infant Pain Profile
RDS: Respiratory distress syndrome
ROP: Retinopathy of prematurity
SIRS: Systemic inflammatory response syndrome
SPIRIT: Standard Protocol Items: Recommendations for Interventional Trials
